# Mesoporous silica loaded with calcitonin gene-related peptide antagonist and *curcumin* alleviate oxidative stress and inflammation in the sciatic nerve

**DOI:** 10.3389/fmolb.2025.1510141

**Published:** 2025-03-24

**Authors:** Yi Zhu, Zhuoliang Zhang, Liangliang Gao, Yue Tian, Xinyu Lu, Yinhong Jiang, Huibin Su, Chengyong Gu, Chenghuan Shi, Lei Wei

**Affiliations:** Department of Anesthesiology, The Affiliated Suzhou Hospital of Nanjing Medical University, Suzhou Municipal Hospital, Gusu School, Nanjing Medical University, Suzhou, Jiangsu, China

**Keywords:** CGRP antagonist, curcumin, mesoporous materials, nanoparticles, neuropathic pain

## Abstract

**Background:**

Neuropathic pain (NP) is a kind of chronic pain that can lead to neurasthenia. The effectiveness of current drug treatment for NP is still unsatisfactory due to its side effects, addiction and withdrawal. In recent years, researchers have begun to develop nano-drug delivery systems for the diagnosis and treatment of NP diseases.

**Methods:**

We developed a disulfide-bonded magnetic mesoporous silica dual-drug delivery system consisting of *curcumin* (*Cur*) and a calcitonin gene-related peptide (CGRP) antagonist (CGRPi), and characterized by electron microscopy, Dynamic Light Scattering (DLS), Zeta, specific surface area and pore size detection. At the cellular level, the biocompatibility of CGRPi@*Cur*@Fe_3_O_4_@mSiO_2_-PEG (FMCC) nanoparticles were tested by CCK-8 and dead/alive staining kit in BV2 cells; Inflammation levels and oxidative stress were measured by enzyme linked immunosorbent assay (ELISA) in lipopolysaccharide (LPS)-induced BV2 neuroinflammation model. *In vivo*, chronic constriction injury (CCI) model was constructed, and the effect of FMCC on pain behavior of CCI mice was detected by von Frey filaments test and thermal hyperalgesia; The effects of FMCC on the anti-inflammatory and oxidative stress of CCI were determined by pathological tests (HE and ROS staining), RT-PCR and ELISA.

**Results:**

FMCC had good biocompatibility and could be taken up by BV2 cells. At the cellular level, FMCC could effectively reverse oxidative stress, inflammation and CGRP expression in LPS-induced neuroinflammation model *in vitro*. At the animal level, the mice with CCI were administered with FMCC, which effectively reduced oxidative stress and inflammation and sustained relief of neuropathic pain.

**Conclusion:**

This study provides a new approach for the treatment of neuropathic pain.

## 1 Introduction

Neuropathic pain is pain caused by damage or disease of the sensory system, which is often manifested as spontaneous persistent pain and hyperalgesia (Rosenberger et al., 2020). Epidemiological studies have shown that the incidence of neuropathic pain in the general population is about 3 %–17% (Cavalli et al., 2019), which brings serious economic and social burden to people and society. At present, the exact pathogenesis of neuropathic pain is still unclear, and the clinical treatment of neuropathic pain is mainly through drug intervention (Finnerup et al., 2021). However, pharmacologic treatment of neuropathic pain, such as nonsteroidal anti-inflammatory drugs and opioids, often has unsatisfactory results (Attal and Bouhassira, 2021). Most patients do not receive satisfactory treatment, and some drugs are often accompanied by various adverse reactions (Macone and Otis, 2018). Therefore, the search for drugs with better efficacy, fewer side effects and affordability in the treatment of neuropathic pain has become one of the hot spots in the research of neuropathic pain.

*Curcumin* (*Cur*) is a hydrophobic acidic compound extracted from the herb Curcuma longa (Hassanzadeh et al., 2020). *Cur* plays an important role in the treatment of a variety of diseases, such as nerve injury, atherosclerosis, malignant tumors, diabetes, autoimmune diseases and neuropathic pain (Kotha and Luthria, 2019). The analgesic effect of *Cur* depends on the comprehensive action of a variety of drug mechanisms, such as: reducing the damage caused by oxidative stress, inhibiting the release of inflammatory cytokines and adhesion molecules, activating glial cells, and inhibiting the activation of protein kinases (Uddin et al., 2021).

The neuropeptide calcitonin gene-related peptide (CGRP) is the main neurotransmitter of C-nerve fibers, which is produced in the central and peripheral nervous system, especially in the spinal cord, dorsal root ganglion and trigeminal ganglion, and plays an important role in the initiation and maintenance of neuropathic pain (Kang and Govindarajan, 2021). Studies have reported that the expression of CGRP is increased after nerve injury and plays an important role in the generation and maintenance of pain behavior (Hegazy et al., 2020). Inhibiting the expression of CGRP can significantly improve the pain behavior after peripheral nerve injury (Paige et al., 2022). *Cur* is a polyphenol compound extracted from the root of turmeric, which has anti-oxidative stress effect. It has been reported to reduce the level of CGPR in DRG neurons and reduce lumbar (Xiao et al., 2017). Although the above studies suggest that *Cur* can be used as an alternative to conventional drugs for the treatment of neuropathic pain, the poor water solubility, poor targeting and poor bioavailability of *Cur* have become difficulties in the development of *curcumin* drugs.

Currently, magnetic targeting offers tantalizing possibilities (Polyak and Friedman, 2009). It was originally developed to optimize chemotherapy procedures for *in vivo* targeting with the assistance of magnetic fields, which would allow a greater proportion of the magnetic nanomaterials to reach the injury site quickly (Bhattacharya et al., 2022). The Fe_3_O_4_ form of iron oxide nanoparticle is approved for clinical applications due to their remarkable biocompatibility (Zhao et al., 2020). As one of the most popular magnetic resonance imaging reagents, superparamagnetic Fe_3_O_4_ nanoparticles are widely used for disease diagnosis (Park et al., 2020). Mesoporous silica nanoparticles (MSNs) have also been shown to deliver drug molecules to target cells and tissues due to their plasticity in varying sizes, shapes, and surface modifications (García-Fernández et al., 2021). Due to its large surface area and mesoporous structure, mesoporous silica can make it possible to deliver different drugs in a multi-stage manner, which may become an ideal method for targeted treatment of chronic pain.

In recent years, MSNs have attracted the interest of many researchers in therapeutic applications as a promising drug carrier due to their unique properties. Uribe Madrid et al. synthesized core-shell nanostructures Fe_3_O_4_@mSiO_2_. The nanostructure has reasonable biocompatibility and good drug release performance. After loading ibuprofen, the drug release amount can reach 81% (Uribe Madrid et al., 2015). Elbialy et al. synthesized MSNs with pH responsive properties, which exhibited high sustained release in the tumor microenvironment after loading *Cur* (Elbialy et al., 2020). Kong et al. encapsulated *Cur* in MSN pores to enhance its antioxidant activity, biocompatibility, and anticancer activity (Kong et al., 2019). Due to the better bioavailability and solubility of *Cur* encapsulated in the pores of MSNs compared to *Cur*, the therapeutic effect of MSNs loaded with *curcumin* is greater than that of *Cur* (Yadav et al., 2019). The polyethylene glycol functionalized MSNs enhances the biocompatibility, stability, and permeability of *curcumin*, prolonging the circulation time of the nanomedicine delivery system in the bloodstream (Park et al., 2009). Chronic constriction injury (CCI) model of the sciatic nerve was first proposed by Bennett and Xie as a model to study neuropathic pain (Bennett and Xie, 1988). It has since been widely used as one of the most commonly used animal models of neuropathic pain.

In this study, we fabricated a disulfide-bonded magnetic mesoporous silica dual drug delivery system (Fe_3_O_4_@mSiO_2_), which allows efficient encapsulation of *Cur* and CGRPi due to the high specific surface area and mesoporous channels of mesoporous silica (Supplementary Figure S1). The safety and efficacy of CGRPi@*Cur*@Fe_3_O_4_@mSiO_2_-PEG (FMCC) nanocomposites in reducing pathological pain, neuroinflammation and oxidative stress *in vitro* and *in vivo* were investigated.

## 2 Materials and methods

### 2.1 Materials

FeCI_2_.4H_2_O (cat: 220299), FeCI_3_.6H_2_O (cat: 236489), NH_3_·H_2_O (28 wt%, cat: 105423), chloroform, cetyltrimethylammonium bromide (CTAB, cat: H6269), ethyl orthosilicate (cat: 131903), ethyl acetate (cat: 650528), methanol (cat: 34860), (3-Mercaptopropyl) trimethoxysilane (MPTMS, cat: 175617), acetone (cat: 179124), cysteamine (cat: 30070), *Curcumin* (cat: C1386), hydrazine (cat: 225819), buffered saline (PBS, cat: P2272), DAPI (cat: D9542), lipopolysaccharide (LPS, cat: L4391) and BSA (cat: V900933) were purchased from Sigma-Aldrich (St. Louis, MO, USA). CGRPi (BIBN4096), dimethyl sulfoxide (DMSO, cat: HY-Y0320), fluorescein 5-isothiocyanate (FITC, cat: HY-66019) and SYBR Green qPCR Master Mix (cat: HY-K0501A) were obtained from MCE (USA). 0.25% pancreatic enzyme (cat: 25200072) and MEM medium (cat: 11095080) were supplied by Gibco (USA). CCK-8 reagent (cat: E-CK-A362) was purchased from Elabscience (China). Calcein/PI solution (cat: C2015S) was obtained from Beyotime Biotechnology (China). Enzyme linked immunosorbent assay (ELISA) kit (cat: mIC50536-1, mIC50274-1, mIC50300-1 and IC50325-1) was from Shanghai Enzyme-linked Biotechnology (China). DCFH-DA (cat: S0033S) was supplied by Beyotime Biotechnology (China). TRIzol Reagent (cat: 15596018CN) was purchased from Invitrogen (Carlsbad, CA, USA). iScript™ cDNA Synthesis Kit (cat: 1708891) was obtained from Bio-Rad Laboratories (USA).

### 2.2 Synthesis of CGRPi@*Cur*@Fe_3_O_4_@mSiO_2_-PEG (FMCC)

Firstly, Fe_3_O_4_ nanoparticles are synthesized. They were prepared by coprecipitation according to the previous research (Albalawi et al., 2021). Briefly, 5.56 g of FeCI_2_.4H_2_O and 10.8 g of FeCI_3_.6H_2_O were dissolved into aqueous solution (100 mL). Then 20 mL of NH_3_·H_2_O was injected into this solution and continued stirring for 1 h at 85°C. Next, the hydrophilic magnetic nanocrystals were collected with magnets and washed with ultra-pure water three times. Finally, the precipitate obtained were dried to obtain magnetic Fe_3_O_4_ nanoparticles.

Then Fe_3_O_4_@mSiO_2_ was synthesized with ferric oxide as substrate. 75 mg of magnetic Fe_3_O_4_ nanoparticles were dispersed into chloroform solution (1 mL), and 0.25 g of CTAB was dissolved in DI water solution (12 mL), followed by mixing and heating to 70°C. Then 250 mL of DI water solution was immediately added to the mixture and mechanically stirred at 40°C for 1 h, followed by rapid addition of 7.5 mL NH_3_·H_2_O, 1.25 mL of ethyl orthosilicate, and 12.5 mL of ethyl acetate and stirring at 40°C at 80 rpm for 6 h. The mixture was washed by centrifugation with water and ethanol for five times, and magnetic Fe_3_O_4_@mSiO_2_ nanoparticles was obtained.

Then, disulfide bond modified Fe_3_O_4_@mSiO_2_ nanoparticles were synthesized on the basis of Fe_3_O_4_@mSiO_2_. Firstly, 250 mg of Fe_3_O_4_@mSiO_2_ nanoparticles was dispersed in 80 mL of methanol, and then 0.7 mL of MPTMS was added into the mixture. The reaction mixtures were stirred for 20 h at room temperature under N_2_ protection, and immediately acetone was added. The Fe_3_O_4_@mSiO_2_-SH nanoparticles was obtained after centrifugation for five times. 0.1 g of Fe_3_O_4_@mSiO_2_-SH was ultrasonic-dispersed in 10 mL methanol and 0.2 g of cysteamine was added, followed by stirring at room temperature for 24 h. The disulfide bond modified Fe_3_O_4_@mSiO_2_ nanoparticles were collected by washing for three times and drying in vacuum oven for 24 h.

Then CGRPi@Fe_3_O_4_@mSiO_2_-PEG (CGRPi@FM) nanoparticles were synthesized on the basis of disulfide bond modified Fe_3_O_4_@mSiO_2_. 10 mg of disulfide bond modified Fe_3_O_4_@ mSiO_2_ and 2 mg CGRP antagonist were added to 1 mL of DMSO. The mixture was stirred in dark place at room temperature overnight. The CGRPi@Fe_3_O_4_@mSiO_2_ nanoparticles were obtained after centrifugation and drying.

10 mg of CGRPi@Fe_3_O_4_@mSiO_2_ nanoparticles were ultrasonic-dispersed into 10 mL of DI water solution. Subsequently, 10 mg DCC, 5 mg DMAP and 25 mg of PEG were added and stirred at room temperature for 24 h. Then 1 mL hydrazine phosphate was added into the above solution and stirred at room temperature for 24 h. The resulting mixture was centrifugally (10,000 rpm, 10 min) separated and then vacuum dried to obtain CGRPi@Fe_3_O_4_@mSiO_2_-PEG (CGRPi@FM-PEG).

Cur@Fe_3_O_4_@mSiO_2_-PEG (Cur@FM) were synthesized on the basis of disulfide bond modified Fe_3_O_4_@mSiO_2_. 10 mg of Fe_3_O_4_@mSiO_2_ nanoparticles were dispersed in 2 mL of DMSO solution containing 2.5 mg of *curcumin*. Then the mixture was stirred in dark place at room temperature for 12 h. Afterwards, the precipitation after centrifugation was *Cur*@Fe_3_O_4_@mSiO_2._ 10 mg of *Cur*@Fe_3_O_4_@mSiO_2_ nanoparticles were ultrasonic-dispersed into 10 mL of DI water solution. Subsequently, 10 mg DCC, 5 mg DMAP and 25 mg of PEG were added and stirred at room temperature for 24 h. Then 1 mL hydrazine phosphate was added into the above solution and stirred at room temperature for 24 h. The resulting mixture was centrifugally (10,000 rpm, 10 min) separated and then vacuum dried to obtain *Cur*@Fe_3_O_4_@mSiO_2_-PEG (*Cur*@FM).

Finally, the FMCC nanoparticles were synthesized on the basis of disulfide bond modified Fe_3_O_4_@mSiO_2_. 10 mg of Fe_3_O_4_@mSiO_2_, 2 mg of CGRPi and 2.5 mg of *Cur* dissolved in 1 mL of DMSO solution. The mixture was stirred in dark place for 24 h. After washed with DI water solution three times, CGRPi@*Cur*@Fe_3_O_4_@mSiO_2_ was obtained. 10 mg of CGRPi@*Cur*@Fe_3_O_4_@mSiO_2_ nanoparticles were dispersed ultrasonically into 10 mL of DI water solution. Subsequently, 10 mg DCC, 5 mg DMAP and 25 mg of PEG were added and stirred at room temperature for 24 h. Then 1 mL hydrazine phosphate was added into the above solution and stirred at room temperature for 24 h. The resulting mixture was centrifugally (10,000 rpm, 10 min) separated and then vacuum dried to obtain FMCC.

### 2.3 Drug loading and release of FMCC

The FMCC nanocomposites were prepared as described above. The resulting mixture was centrifugally (10,000 rpm, 10 min) separated and then vacuum dried to obtain FMCC. The supernatant was detected at 426 and 285 nm by spectrophotometer (SpectraMax iD3, Molecular Devices, USA). The concentrations of *Cur* and CGRPi in the supernatant were calculated using the standard curve. The amount of drug encapsulated was obtained by the difference between the initial amount of drug added and the amount of drug remaining in the supernatant. Drug loading efficiency (%) was calculated using the following equation. Drug loading efficiency (%) = Amount of drug encapsulated (mg)/(weight of FMCC) × 100.

For *Cur* and CGRPi release studies, 1 mg of FMCC was dispersed in 50 mL of PBS solution. The resulting mixture was stirred regularly at 100 rpm and 37°C. While mixing was in progress, samples were taken from the drug release medium at regular intervals to determine the amount of drug released. For this, the sample was first centrifuged at 12,000 rpm for 15 min. The sample was detected at 426 nm by spectrophotometer. The concentrations of *Cur* in the sample were calculated using the standard curve. After reading, the sample was returned to the release medium. The amount of drug released was calculated with the help of a standard calibration curve. The same method given above was followed for the CGRPi release from FMCC. The absorbance at 285 nm was detected by spectrophotometer and corrected by the standard curve method. The concentrations of CGRPi in the sample were calculated using the standard curve.

### 2.4 Characterization

SEM images were captured by scanning electron microscopy (JEM100CXⅡ, Japan) at 1.0 kV. TEM images were captured by electron microscopy (LVEM5, USA) at 5 kV. Sample preparation: add 5 mg nanoparticles to ethanol, ultrasonic for 5 min. Then they were added to the copper mesh and dried for testing.

The measurement of particle sizes and zeta potential were performed by a nanometer particle size analyzer (Nanotrac Flex, Germany).

The specific surface area and pore size distribution of the samples were detected by an automatic gas adsorption instrument (BELSORP HP, Japan) and analyzed by Brunauer-Enmett-Teller (BET) and Barrett-Joyner-Halenda (BJH) methods.

### 2.5 BV2 cell culture

BV2 cells were obtained from American Type Culture Collection (ATCC, Rockville, MD, USA) and maintained in MEM supplemented with 10% FBS in a humidified incubator containing 5% CO_2_ at 37°C.

### 2.6 CCK-8 assay

5 × 10^3^ BV2 cells were inoculated on 96-well plates and incubated overnight in an incubator at 37°C and 5% CO_2_. The cells were treated with different concentrations of FMCC (0, 1, 5, 10, 20 μg/mL) for 24 h in the presence or absence of LPS. 10 μL CCK8 reagent was added to incubate in the incubator for 2 h, and the absorbance value was detected at 450 nm.

### 2.7 Dead-living cell staining

2 × 10^5^ BV2 cells were inoculated on 12-well plates and incubated overnight in an incubator at 37°C and 5% CO_2_. The cells were treated with 20 μg/mL Fe_3_O_4_@mSiO_2_ and FMCC for 6 h. Then Calcein/PI solution were added to incubate at 37°C in dark place, washed with PBS for three times and observed with microscope (Leica DMi8, Germany). Fluorescence intensities were measured using a fluorescence microscope, with calcein detected at excitation/emission wavelengths of 494/517 nm (blue filter) and propidium iodide (PI) detected at 535/617 nm (green filter).

### 2.8 Cellular uptake assay

1 × 10^5^ BV2 cells were inoculated on 24-well plates and incubated overnight in an incubator at 37°C and 5% CO_2_. The cells were treated with 20 μg/mL Fe_3_O_4_@mSiO_2_-FITC. Fe_3_O_4_@mSiO_2_-FITC was obtained by reacting Fe_3_O_4_@mSiO_2_ with FITC under dark condition for 12 h. At 2 and 6 h, DAPI was added to stain 10 min, washed with PBS for three times and observed with microscope. Fluorescence intensities were measured using a white light laser confocal microscope (Leica STELLARIS, Germany), with FITC detected at excitation/emission wavelengths of 490/520 nm and DAPI at 358/461 nm. The ImageJ software was used for quantitative analysis (Jensen, 2013).

### 2.9 *In vitro* neuroinflammatory model

BV2 cells were treated with 100 ng mL^−1^ LPS for 24 h to establish an *in vitro* neuroinflammatory model. Then, BV2 cells were divided into five groups: 1) Control group; 2) LPS group; 3) LPS + CGRPi@FM group; 4) LPS + *Cur*@FM group; 5) LPS + FMCC group.

### 2.10 Chronic constriction injury (CCI) model

6–8 weeks-old, 18–22 g healthy C57BL/6 mice were purchased by SPF Biotechnology Co.,Ltd. CCI surgery was performed in accordance with previous study (Zhang et al., 2019). The mice were anesthetized by inhaling 4% isoflurane. The right thigh hair of the mouse was scraped and the left biceps femoris was bluntly separated to expose the sciatic nerve. The sciatic nerve was partially ligated at four points using 4-0 colored sutures, with each ligature placed approximately 1 mm apart proximal to the trifurcation site. After that, the muscles and skin were treated with iodine solution. In the sham operation group, the operation method was the same except that the sciatic nerve was not lapped. After CCI surgery, a small magnet was sterilized and fixed to the skin at the surgical site.

### 2.11 *In vivo* experiment design

25 C57BL/6 mice were divided into five groups 7 days after CCI surgery:1) Control group: the sham operation group; 2) CCI group; 3) CCI + CGRPi@FM group; 4) CCI + *Cur*@FM group; 5) CCI + FMCC group. In the treatment group, 120 mg/kg nanoparticles were injected through the tail vein on day 1 and day 6. After treatment, the threshold of paw withdrawal threshold was detected by using the mesh to stimulate the right plantar of mice with von Frey fibers. And the paw withdrawal latency was detected by stimulating the right plantar of mice with heat. Then sciatic nerve was taken and the therapeutic effect of each group was observed by H&E staining.

### 2.12 Enzyme linked immunosorbent (ELISA) assay

BV2 cells were treated with LPS for 24 h and then treated with 20 μg/mL CGRPi@FM, *Cur*@FM, FMCC, or PBS for 24 h. The cell supernatant of each group was collected. *In vivo*, blood was collected from the orbital vein after treatment. The samples were allowed to clot for 30 min at room temperature and then centrifuged to isolate the supernatant. Then the content of TNF-α, IL-1β, IL-6 and IL-10 content was detected by Mouse TNF-α ELISA kit, Mouse IL-1β ELISA kit, Mouse IL-6 ELISA kit and Mouse IL-10 ELISA kit.

### 2.13 Detection of oxidative stress indexes

BV2 cells were treated with LPS for 24 h and then treated with 20 μg/mL CGRPi@FM, *Cur*@FM, FMCC, or PBS for 24 h. The cell of each group was collected and malondialdehyde (MDA), superoxide dismutase (SOD), catalase (CAT) and glutathione-peroxidase (GSH-Px) content was detected by ELISA kit.

### 2.14 Reactive oxygen species (ROS) detection

BV2 cells were treated with LPS for 24 h and then treated with 20 μg/mL CGRPi@FM, *Cur*@FM, FMCC, or PBS for 24 h. Then the DCFH-DA were added to incubate for 30 min. And the ROS signals were observed with microscope. Fluorescence intensities were measured using a fluorescence microscope at excitation/emission wavelengths of 488/525 nm (blue filter). The ImageJ software was used for quantitative analysis.

The mice in each group were euthanized after treatment. The sciatic nerve was quickly extracted and frozen with liquid nitrogen. The ROS content was detected by DHE probe method and photographed by microscope. Fluorescence intensities were measured using a fluorescence microscope at excitation/emission wavelengths of 535/610 nm (green filter).

### 2.15 RT-PCR assay

The total RNA was extracted from sciatic nerve using TRIzol Reagent. The cDNA was synthesized using iScript™ cDNA Synthesis Kit. The qPCR was performed on ABI 7500-Fast Real-Time PCR System (Applied Biosystem, Foster City, CA, USA) using SYBR Green qPCR Master Mix and the β-actin was used as an internal control. The primer sequence is shown in Table 1.

**TABLE 1:** The primer sequences of TNF-α, IL-1β, IL-6, IL-10 and β-actin.

Gene name	Forward 5′-3′	Reverse 5′-3′
TNF-α	CACAGAAAGCATGATCCGCG	GTGCCCTCTGTGCTTGATCT
IL-1β	CCCATGTTGTAGTGACCCCC	CATGGTTGGGCTTGGGAGTG
IL-6	TGAGAGAGGAGTGTGAGGCA	ACAGAGAATGGCCCACTGTG
IL-10	CAGAGTGTGGCAGTGGGAAT	TGCTTCAAACCCCCAAACCT
β-actin	CTTCGCTCTCTCGTGGCTAG	AAGAGGGGGAGAGGAAGAGC

### 2.16 Biosafety assay

6–8 weeks-old, 18–22 g of healthy C57BL/6 mice were given a single dose of FMCC 120 mg/kg via the tail vein injection. On 0 day, 3 days and 30 days, blood was then taken from the ocular vein for routine blood test and biochemical analysis. Routine blood test and biochemical analysis were performed by a fully automated biochemical analyzer (Indiko™ Plus, Thermo Scientific, USA). The heart, liver, spleen, lung and kidney were extracted to H&E staining.

### 2.17 Statistical analysis

Data are presented as the mean ± standard error of mean (SEM) from at least three separate experiments. Statistical analysis was performed using GraphPad Prism statistical software. Comparisons between two groups were performed using Student’s t-test. Comparisons among multiple groups were analyzed using one–way ANOVA, with Bonferroni correction applied. The differences were deemed statistically significant at *p* < 0.05.

## 3 Results and discussion

### 3.1 Characterization of FMCC drug delivery system

**FIGURE 1:**
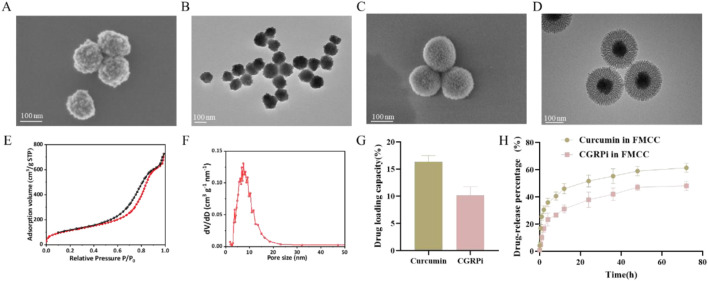
Characterization of Fe_3_O_4_, Fe_3_O_4_@mSiO_2_ and FMCC nanoparticles. **(A)** SEM images of Fe_3_O_4_ nanoparticles; **(B)** TEM images of Fe_3_O_4_ nanoparticles. **(C)** SEM images of Fe_3_O_4_@mSiO_2_ nanoparticles; **(D)** TEM images of Fe_3_O_4_@mSiO_2_ nanoparticles; **(E)** Nitrogen adsorption-desorption isotherm of Fe_3_O_4_@mSiO_2_ nanoparticles. The adsorption volume (cm^3^/g STP) of N_2_ gas was measured as a function of relative pressure (P/P_0_, where P_0_ is the saturation pressure of N_2_ at 77 K). The specific surface area (427 m^2^/g) was calculated from the linear region of the BET plot based on the BET theory; **(F)** Pore size distribution (dV/dD) of Fe_3_O_4_@mSiO_2_ nanoparticles was analyzed using the BJH method, showing a predominant pore size of ∼7.4 nm. **(G)** The drug loading capacity of *Cur* and CGRP antagonist on Fe_3_O_4_@mSiO_2_-PEG; **(H)** Plots of the release curves of the drugs *Cur* and CGRP antagonist from the FMCC nanocomplex in PBS buffer.

As reported the core-shell nanostructures Fe_3_O_4_@mSiO_2_ has been shown reasonable biocompatibility and good drug release performance (Zhang et al., 2021). Fe_3_O_4_@mSiO_2_ is a suitable drug delivery carrier, and it also possesses functionalities such as magnetic targeting, magnetic resonance imaging. Curcumin has been reported to produce a healing analgesic effect in chronic neuropathic pain (Caillaud et al., 2020). In addition, CGRP expression levels are elevated after nerve injury and play an important role in generating and maintaining pain behavior, and inhibiting CGRP expression can significantly improve pain behavior after peripheral nerve injury (Benarroch, 2011). Therefore, in this study, we have synthesized Fe_3_O_4_@mSiO_2_ with novel structure as the combined delivery carrier for curcumin and CGRPi. Firstly, Fe_3_O_4_ nanoparticles synthesized by coprecipitation method were extensively characterized. The SEM images showed that it was spherical, with a particle size of about 110 nm (Figure 1A). TEM images showed that Fe_3_O_4_ nanoparticles were round, well dispersed, and relatively uniform in particle size, mainly about 110 nm (Figure 1B). Dynamic light scattering (DLS) results (Supplementary Figure S2A) showed an increase in the hydrodynamic size of Fe_3_O_4_ nanoparticles to 122.4 nm.

Further, mesoporous silicon dioxide (mSiO_2_) was grown on the surface of Fe_3_O_4_ nanoparticles to synthesize the shell karyotype nanoparticles Fe_3_O_4_@mSiO_2_. Furthermore, since disulfide bonds in the nanocore framework are one of the most efficient cleavable joints in biology, they can be degraded and cleaved in the presence of glutathione (Fu et al., 2022). Therefore, cysteamine is used to make mSiO_2_ doped with disulfide bonds. Then, the Fe_3_O_4_@mSiO_2_ nanoparticles were characterized by electron microscopy, DLS, Zeta potential analysis and nitrogen isothermal adsorption-desorption curves. It can be observed from SEM (Figure 1C) and TEM (Figure 1D) that the length of mSiO_2_ nanorods is about 60 nm, and mesoporous pores are obvious and uniform, and they grow evenly on the surface of Fe_3_O_4_, completely enveloping Fe_3_O_4_ nanospheres. The morphology of Fe_3_O_4_ nanospheres remained intact. High specific surface area and large pore volume can effectively adsorb and load drugs. Therefore, the specific surface area and pore size of Fe_3_O_4_@mSiO_2_ were measured by nitrogen isothermal adsorption-desorption curve. The synthesized Fe_3_O_4_@mSiO_2_ showed a typical type IV isotherm and hysteresis ring. This is caused by the ordered mesopore in the mSiO_2_ nanorods (Figure 1E). Fe_3_O_4_@mSiO_2_ has a Brunauer-Emmett-Teller (BET) surface area and a Barrett-Joyner-Halenda (BJH) pore size of 427 m^2^/g and ∼7.4 nm, respectively (Figure 1F). In addition, the hydrated particle size of Fe_3_O_4_@mSiO_2_ measured by DLS was 220.2 nm (Supplementary Figure S2B), and the hydrated particle size of FMCC was 240.2 nm (Supplementary Figure S2C). The structure of the nanoparticles loaded with *Cur* and CGRPi was not altered as observed by TEM (Supplementary Figure S2D), and the BJH pore size was reduced to ∼6.3 nm (Supplementary Figure S2E). The Zeta surface potentials of Fe_3_O_4_, Fe_3_O_4_@mSiO_2_, Fe_3_O_4_@mSiO_2_ loaded with *Cur* and CGRP antagonists, and FMCC were −19.2, −16.5, −15.5, and −22.9 mV, respectively (Supplementary Figure S2F).

The loading capacities of *Cur* and CGRPi on FMCC were 16.4% and 10.2%, respectively (Figure 1G). To investigate the kinetics of drug release from the nanocomposites in detail, we tracked the release of the drugs *Cur* and CGRPi from FMCC in PBS solution over time. Under the experimental conditions (PBS solution, pH7.4), both drugs demonstrated slow release (Figure 1H). In this regime, the rate of *Cur* release increased slightly compared to CGRPi. Although the release rates of *Cur* were only slightly higher than those of CGRPi, the actual release amounts of *Cur* were approximately twice that of CGRPi, due to the higher initial loading of *Cur* in the nanoparticles. The differences in release efficiency and drug loading between *Cur* and CGRPi can be attributed to several factors. First, the molecular properties of the drugs, such as solubility, hydrophobicity, and molecular size, significantly influence their interaction with the nanocarrier and subsequent release behavior. *Cur* is a hydrophobic molecule with a relatively small molecular size, which facilitates its encapsulation and sustained release from the hydrophobic core of the nanoparticle (Wang et al., 2020). In contrast, CGRPi, being a peptide, has higher hydrophilicity and larger molecular size, which may limit its loading efficiency and result in slower release kinetic (Luo et al., 2023). Second, the chemical interactions between the drugs and the nanocarrier material play a crucial role. *Cur* can form strong π-π interactions and hydrogen bonds with the silica matrix of the nanoparticles, leading to higher loading capacity and controlled release (Lin et al., 2022). On the other hand, CGRPi may rely more on electrostatic interactions or physical entrapment, which are less stable and result in lower loading efficiency and faster initial release (Liang et al., 2020).

### 3.2 The safety properties and cellular internalization of FMCC *in vitro*

**FIGURE 2:**
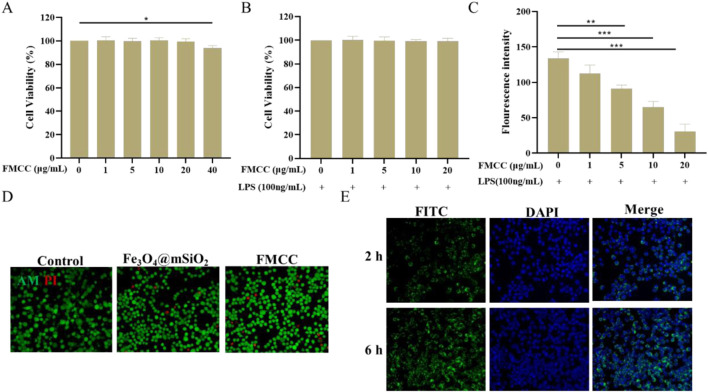
The safety properties and cellular internalization of FMCC *in vitro*. **(A)** Effects of different concentrations (0, 1, 5, 10, 20, and 40 μg/mL) of FMCC alone on BV2 cell viability; **(B)** LPS-induced BV2 cells were pretreated with different concentrations (0, 1, 5, 10, and 20 μg/mL) of FMCC for 24 h. Cell viability was determined using CCK-8 assay; **(C)** Detection of reactive oxygen species (ROS) in LPS-induced BV2 cells using the DCFH-DA kit. LPS-induced BV2 cells were treated with different concentrations (0, 1, 5, 10, and 20 μg/mL) of FMCC for 24 h. ROS levels were measured by fluorescence intensity, which correlates with the oxidation of DCFH-DA to DCF; **(D)** Dead and alive staining of BV2 cells after co-incubation with Fe_3_O_4_@mSiO_2_ or FMCC; **(E)** Images of BV2 cells internalization after co-cultured with Fe_3_O_4_@mSiO_2_-FITC nanoparticles for 2 h and 6 h. n = 3. Data are presented as the mean ± standard error of mean.

Current studies have shown that CGRP content increases in inflammatory pain and neuropathic pain, and the upregulation of CGRP level contributes to the enhancement of pain perception, which is the target of analgesic treatment (Johanes et al., 2020). Prior to this, the toxicity of FMCC on BV2 cells was first investigated using CCK-8 assay for a period of 24 h at different concentrations. As shown in Figure 2A, the concentrations of FMCC less than 20 μg/mL did not induce any detectable cytotoxicity, whereas cytotoxicity was induced at concentrations of 40 μg/mL. Next, we treated LPS-induced BV2 cells with different concentrations of FMCC for their cytotoxic effects. Different concentrations of FMCC up to 20 μg/mL had no significant toxic effect on BV2 cell viability (Figure 2B). The ROS fluorescence was used to further detect the effect of different concentrations of FMCC on the ROS level of BV2 cells induced by LPS. With the increase of FMCC concentration, the intracellular ROS level in BV2 cells gradually decreased and reached the lowest level at 20 μg/mL (Figure 2C). These results indicate that the incubation of FMCC significantly reduces LPS-induced ROS production in BV2 cells without cytotoxicity, which would potentially alleviate inflammatory pain and neuropathic pain. In subsequent experiments, we chose FMCC at a concentration of 20 μg/mL for subsequent cell experiments.

Based on this, cell viabilities and cellular internalization of FMCC were studied BV2 cells. As shown in Figure 2D, even the incubated concentration set as high as 20 μg/mL and the co-culture time extended as long as 24 h, the cell viabilities of BV2 cells remained over 90%, pronouncedly confirming that the FMCC nanocomposites possessed perfect biocompatibility. Further, Figure 2E and Supplementary Figure S3 showed the images and quantitative fluorescence intensities of BV2 cells after incubated with FMCC for 2 h and 6 h, respectively. This nanocomposite material showed a green light, and the intensity of the fluorescence becomes stronger with the increase of time. These results indicate that FMCC can effectively carry drugs into glial cells, consistent with previous reports that Fe_3_O_4_@mSiO_2_ promotes drug uptake by cells (Sun et al., 2020). Efficient cellular uptake and drug release may be critical for pain relief by FMCC.

### 3.3 Effects of FMCC on inflammatory factors, oxidative stress and reactive oxygen species (ROS) in LPS-induced BV2 cells *in vitro*

**FIGURE 3:**
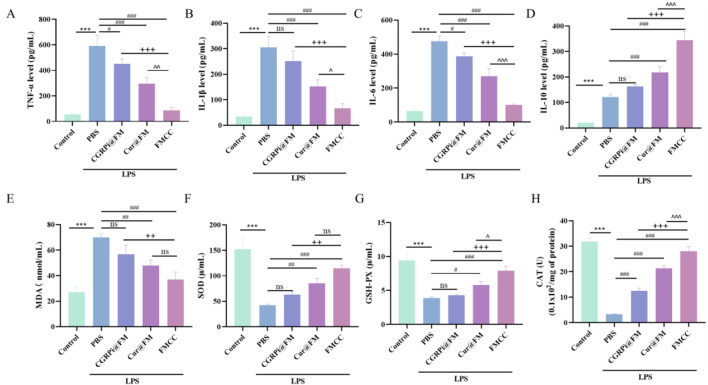
Effects of FMCC on inflammatory factors and oxidative stress in LPS-induced BV2 cells *in vitro*. **(A–D)** TNF-α, IL-1β, IL-6 and IL-10 content in the supernatant of BV2 cells treated with LPS, CGRPi@FM, *Cur*@FM, and FMCC were detected by ELISA analysis; **(E–H)** MDA, SOD, GSH-PX, and CAT content in BV2 cells treated with LPS, CGRPi@FM, *Cur*@FM, and FMCC were detected by ELISA analysis. ns *P* > 0.05 no significant difference. n = 3. Data are presented as the mean ± standard error of mean.

It is well known that neuropathic pain induced by chronic constriction injury (CCI) elicits a persistent inflammatory response (Liu P. et al., 2023). Tumor Necrosis Factor-α (TNF-α) appears as the earliest cytokine in the inflammatory cascade and reaches its peak at an early stage (Jang et al., 2021). TNF-α and IL-1β, as early proinflammatory factors, have been well validated in peripheral and central sensitization of neuropathic pain (Boakye et al., 2021). To determine whether FMCC nanocomposites could protect BV2 cells from inflammatory injury, we used LPS treatment of BV2 cells as an *in vitro* model of neuroinflammation and compared the effects of nanoparticles loading two drugs or a single drug. Upon LPS stimulation, cells increased the production of pro-inflammatory cytokines, including TNF-α, IL-1β, and Interleukin-6 (IL-6), and the anti-inflammatory cytokine Interleukin-10 (IL-10). Direct treatment with *Cur*@FM or CGRPi@FM decreased TNF-α, IL-1β, and IL-6 secretion (Figures 3A–C) and increased IL-10 production (Figure 3D). Notably, a more potent effect was observed in cells treated with FMCC nanocomplexes, suggesting additive or synergistic pharmacological effects of the two loaded drugs. The intracellular CGRP mRNA expression was further detected by RT-PCR assay (Supplementary Figure S4). Consistent with the expected results, LPS treatment enhanced the expression level of CGRP, which was significantly downregulated by FMCC nanocomposites.

**FIGURE 4:**
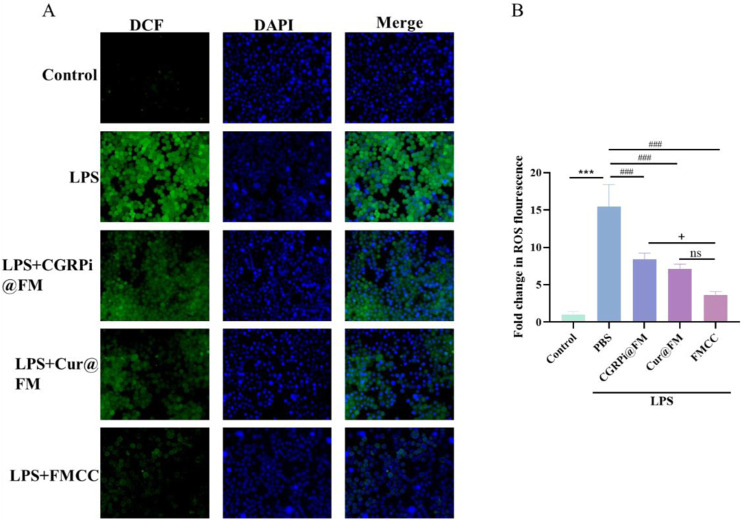
Effects of FMCC on ROS in LPS-induced BV2 cells *in vitro*. DCFH-DA kit detection of ROS in BV2 cells treated with LPS, CGRPi@FM, *Cur*@FM, and FMCC; **(B)** Fluorescence semi-quantitative statistics of **(A)**. ns *P* > 0.05 no significant difference. n = 3. Data are presented as the mean ± standard error of mean.

The neuroinflammatory process after nerve injury releases a variety of cytokines, which in turn promotes the occurrence of oxidative stress and the increase of reactive oxygen species, which is also considered to be an important factor in stimulating inflammation (Fakhri et al., 2022). Therefore, research on inhibiting oxidative stress and ROS after nerve injury can play a role in the treatment of neuropathic pain. To explore the effects of LPS induction and FMCC treatment on oxidative stress level and ROS in BV2 cells, we used MDA, SOD, CAT and GSH-Px assay kits to determine changes in the activities of MDA, SOD, GSH-Px, and CAT in BV2 cells after LPS induction and different treatments. Figures 3E–H showed that LPS induced significant increases in MDA and decreases in SOD, GSH-PX and CAT in BV2 cells. CGRPi@FM and *Cur*@FM were able to inhibit the increase of MDA and the decrease of SOD, GSH-PX and CAT, and the inhibitory effect of FMCC was more significant. DCFH-DA kit results showed that LPS induced a significant increase in ROS production in BV2 cells, which was inhibited by CGRPi@FM and *Cur*@FM, and the inhibitory effect of FMCC was more significant (Figures 4A, B). The above results confirmed that FMCC could inhibit LPS-induced oxidative stress and ROS production in BV2 cells.

The above results indicate that inhibiting CGRP activity can effectively suppress the inflammatory response of glial cells and the increase of ROS caused by inflammation. The anti ROS generation and anti-inflammatory effects of curcumin may come from its regulatory role in validating signaling pathways and the inhibitory activity of CGRP (Liu et al., 2023b), but the latter effect still needs further investigation. The above results also indicate that the dual drug FMCC of curcumin and CGRPi can exert the combined anti-inflammatory and ROS effects of the two drugs, enhancing the therapeutic effect.

### 3.4 FMCC nanocomposites alleviated CCI-induced neuropathic pain, inflammation and oxidative stress *in vivo*

**FIGURE 5:**
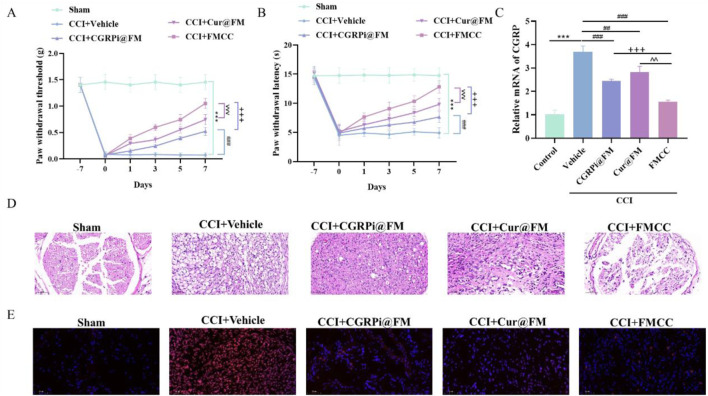
FMCC alleviated CCI-induced neuropathic pain *in vivo*. **(A)** Neuropathic pain was assessed by von Frey filaments test; **(B)** Thermal hyperalgesia was assessed by a radiant heat test; **(C)** RT-PCR detection of CGRP mRNA expression levels in CCI model mice after CGRPi@FM, *Cur*@FM, and FMCC treatment; **(D)** Sciatic nerve sections were stained with hematoxylin and eosin; **(E)** sciatic nerve sections were stained with DHE kit. n = 5. Data are presented as the mean ± standard error of mean.

To elucidate the *in vivo* effects of the FMCC drug delivery system, we generated a mouse model of chronic constriction injury (CCI) of the sciatic nerve (Fonseca-Rodrigues et al., 2021). CGRPi@FM, *Cur*@FM and FMCC were injected into the tail vein of CCI mice on the first day and the sixth day after successful modeling. At the same time, in order to make the nanocomposite magnetic targeting, a strong neodymium magnet was placed at the sciatic nerve injury site. Pain behavior was examined within 2 weeks after surgery (Figures 5A, B). CCI resulted in chronic pain in mice as indicated by increased thermal hyperalgesia and mechanical hyperalgesia compared to the sham-operated group. In contrast, CGRPi@FM, *Cur*@FM and FMCC nanocomposites significantly attenuated neuropathic pain responses, with FMCC having the most significant effect. RT-PCR assay was used to detect the mRNA expression of CGRP in the injured sciatic nerve tissue. The results were consistent with the cellular level (Figure 5C). The cross-sectional sections of sciatic nerve tissue in each group were further stained with hematoxylin-eosin to examine whether FMCC alleviated the histological changes in the sciatic nerve. Compared with the sham group, in the CCI group, the intercellular space was enlarged and the cell arrangement was disordered, which were effectively alleviated by FMCC (Figure 5D). In addition, ROS was significantly increased in the CCI group, while FMCC significantly abolished ROS production (Figure 5E).

**FIGURE 6:**
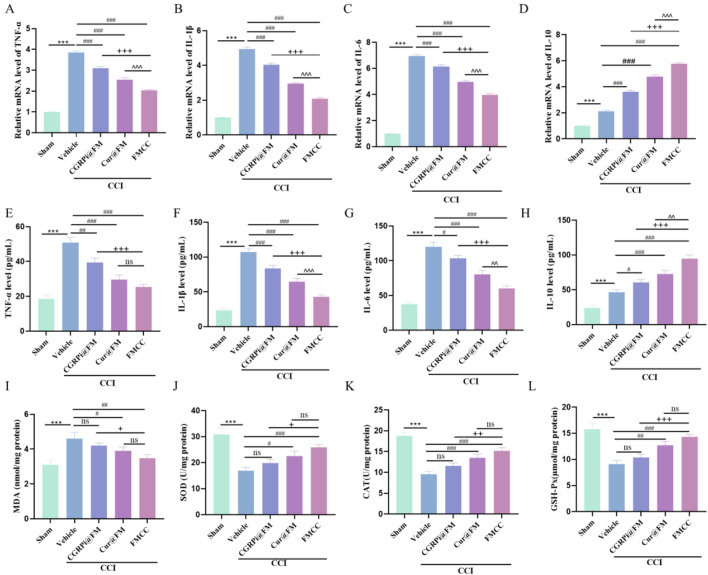
FMCC alleviated CCI-induced inflammation and oxidative stress *in vivo*. **(A–D)** TNF-α, IL-1β,IL-6 and IL-10 mRNA levels in the sciatic nerve of mice and CGRPi@FM, *Cur*@FM, and FMCC treatment were analyzed by RT-PCR analysis; **(E–H)** TNF-α, IL-1β,IL-6 and IL-10 content in the sciatic nerve of mice and CGRPi@FM, *Cur*@FM, and FMCC treatment were detected by ELISA analysis; **(I–L)** MDA, SOD, GSH-PX, and CAT content in the sciatic nerve of mice and CGRPi@FM, *Cur*@FM, and FMCC treatment were detected by ELISA analysis. ns *P* > 0.05 no significant difference. n = 3. Data are presented as the mean ± standard error of mean.

Both RT-PCR and ELISA results showed the ability of FMCC nanocomposites to inhibit inflammation *in vivo*. CCI stimulated the expression of proinflammatory cytokines (TNF-α, IL-1β, and IL-6), on the other hand, the CCI-induced increase in the anti-inflammatory cytokine IL-10 was further enhanced by CGRPi@FM and *Cur*@FM. The FMCC nanocomposite had the most pronounced effect, as shown by the greatest decrease in proinflammatory cytokines and the greatest increase in anti-inflammatory cytokine (Figures 6A–H). Finally, the oxidative stress indicators (MDA, SOD, GSH-Px and CAT) were detected by the kit. Compared with the sham operation group, MDA in the CCI group was significantly increased, and SOD, GSH-PX and CAT were significantly decreased; CGRPi@FM and *Cur*@FM alleviated this phenomenon, with FMCC having the most significant effect (Figures 6I–L).

The pathogenesis of neuropathic pain is complex. In addition to the inflammatory responses are involved in the pathogenesis of neuropathic pain, the dorsal root ganglion (DRG) neurons sense pain and transmit it to the central nervous system, which is involved in the occurrence and maintenance of neuropathic pain (Martin et al., 2019). Lim et al. found that japonica rice leaf extract may relieve CCI-induced neuropathic pain by activating MAPK in DRG and microglia in the spinal cord (Lim et al., 2022). Chu et al. found that atorvastatin may inhibit neuroinflammation in rats with chronic systolic injury by down-regulating dorsal root ganglia and spinal cord nuclear NF-κB (Chu et al., 2015). It was found that electroacupuncture alleviated neuropathic pain by promoting autophagy of dorsal root ganglion macrophages mediated by AMPK/MTOR (Xu et al., 2022). In addition, montelukast can effectively reduce neuropathic pain in CCl rats by inhibiting the activation of p38MAPK and NF-kB signaling pathways in spinal microglia (Zhou et al., 2014).

These results indicate that FMCC can effectively enter the site of sciatic nerve injury in mice, delivering the loaded curcumin and CGRPi to damaged nerve cells, alleviating chronic sciatic nerve injury, inflammatory response, and neuropathic pain response in mice. This is because FMCC can leverage the magnetic targeting effect of Fe_3_O_4_@mSiO_2_ to targeted delivery the curcumin and CGRPi to the damaged nerve area under the influence of an external magnetic field, and treats sciatic nerve pain through the combined action of curcumin and CGRPi. Liang et al. (Liang et al., 2025) synthesized mesoporous silica nanoparticles (MSNs) coated with Fe_3_O_4_ as a drug carrier, which is similar to our nanoparticle design. They loaded the nanoparticles with RADA16-I/RAD-RGI peptide (PD) to construct a neurotrophic microenvironment for the treatment of peripheral nerve defects. Additionally, they functionalized the MSNs with the neurotargeting peptide HLNILSTLWKYR (PT) to enhance targeting efficiency. While their study focused on peripheral nerve regeneration, our work explores the application of Fe_3_O_4_@ mSiO_2_ for drug delivery in neuropathic pain, highlighting the versatility of this nanoparticle platform in different biomedical contexts.

### 3.5 Biocompatibility assessment of FMCC *in vivo*

Additionally, *in vivo* biocompatibility of FMCC was determined. After intravenous injection of our nanoparticles for 3 days and 30 days, blood samples and major organ tissue were collected. Blood bio-chemical tests and H&E staining studies were conducted for exploring the biosafety of FMCC *in vivo*. Here, the serum bio-chemistry factors include alanine transaminase (ALT, Supplementary Figure S5A), aspartate transaminase (AST, Supplementary Figure S5B), serum creatinine (CRE, Supplementary Figure S5C), blood urea nitrogen (BUN, Supplementary Figure S5D), white blood cell (WBC, Supplementary Figure S5E), and Platelet (PLT, Supplementary Figure S5F). Collectively, in comparison with control group (0 day), there were insignificant discrepancy in these indexes of the FMCC based groups at 3 days and 30 days post-administration. In the bargain, the H&E-stained-images of the main normal organ tissues (including heart, liver, spleen, lung and kidney) revealed no apparent pathological injury or normal cellular damage were discovered (Supplementary Figure S5G), preliminarily illustrating the excellent *in vivo* histocompatibility of FMCC as well as its potent bio-application for neuropathic pain patients. These data indicate that FMCC is a nanomedicine with good biocompatibility and *in vivo* targeting. It can simultaneously load two or more drugs and deliver them to the damaged nerve site under the action of an external magnetic field, exerting a combined therapeutic effect. At the same time, it also indicates that the combination of curcumin and CGRPi has a significantly better therapeutic effect on neuropathic pain than the use of the two drugs alone. Through *in vitro* and *in vivo* experiments, we have provided evidence for its feasibility in treating neuropathic pain, which may help promote the development of effective treatments for chronic pain.

## 4 Conclusion

In this study, we successfully designed and synthesized a magnetically targeted nanocomposite that can target the injured area of the sciatic nerve and achieve multifunctional analgesia for the delivery of a single or two analgesic drugs. Our results show that this disulfide bonded magnetic mSiO_2_ nanoparticles can effectively load *Cur* and CGRPi and deliver them to the sciatic nerve injury area. Under conditions of elevated GSH, the disulfide bonds are cleaved, and PEG modification facilitates the complete release of the therapeutic payloads. Through *in vitro* and *in vivo* experiments, we provided evidence for the feasibility of its application in the treatment of neuropathic pain, which may help facilitate the development of effective treatments for chronic pain.

## Data Availability

The raw data supporting the conclusions of this article will be made available by the authors, without undue reservation.

## References

[B1] Albalawi A. E. Khalaf A. K. Alyousif M. S. Alanazi A. D. Baharvand P. Shakibaie M. (2021). Fe3O4(@)piroctone olamine magnetic nanoparticles: synthesize and therapeutic potential in cutaneous leishmaniasis. Biomed. Pharmacother. 139, 111566. 10.1016/j.biopha.2021.111566 33839494

[B2] Attal N. Bouhassira D. (2021). Advances in the treatment of neuropathic pain. Curr. Opin. Neurol. 34 (5), 631–637. 10.1097/wco.0000000000000980 34310363

[B3] Benarroch E. E. (2011). CGRP: sensory neuropeptide with multiple neurologic implications. Neurology 77 (3), 281–287. 10.1212/WNL.0b013e31822550e2 21768598

[B4] Bennett G. J. Xie Y. K. (1988). A peripheral mononeuropathy in rat that produces disorders of pain sensation like those seen in man. Pain 33 (1), 87–107. 10.1016/0304-3959(88)90209-6 2837713

[B5] Bhattacharya S. M K. R. Priyadarshani J. Ganguly R. Chakraborty S. (2022). Targeting magnetic nanoparticles in physiologically mimicking tissue microenvironment. ACS Appl. Mater Interfaces 14 (28), 31689–31701. 10.1021/acsami.2c07246 35786842

[B6] Boakye P. A. Tang S. J. Smith P. A. (2021). Mediators of neuropathic pain; focus on spinal microglia, CSF-1, BDNF, CCL21, TNF-α, wnt ligands, and Interleukin 1β. Front. Pain Res. (Lausanne). 2, 698157. 10.3389/fpain.2021.698157 35295524 PMC8915739

[B7] Caillaud M. Aung Myo Y. P. Mckiver B. D. Osinska Warncke U. Thompson D. Mann J. (2020). Key developments in the potential of curcumin for the treatment of peripheral neuropathies. Antioxidants (Basel) 9 (10), 950. 10.3390/antiox9100950 33023197 PMC7600446

[B8] Cavalli E. Mammana S. Nicoletti F. Bramanti P. Mazzon E. (2019). The neuropathic pain: an overview of the current treatment and future therapeutic approaches. Int. J. Immunopathol. Pharmacol. 33, 2058738419838383. 10.1177/2058738419838383 30900486 PMC6431761

[B9] Chu L. W. Chen J. Y. Wu P. C. Wu B. N. (2015). Atorvastatin prevents neuroinflammation in chronic constriction injury rats through nuclear NFκB downregulation in the dorsal root ganglion and spinal cord. ACS Chem. Neurosci. 6 (6), 889–898. 10.1021/acschemneuro.5b00032 25874913

[B10] Elbialy N. S. Aboushoushah S. F. Sofi B. F. Noorwali A. J. M. Materials M. (2020). Multifunctional curcumin-loaded mesoporous silica nanoparticles for cancer chemoprevention and therapy. Microporous Mesoporous Mat. 291, 109540. 10.1016/j.micromeso.2019.06.002

[B11] Fakhri S. Piri S. Moradi S. Z. Khan H. (2022). Phytochemicals targeting oxidative stress, interconnected neuroinflammatory, and neuroapoptotic pathways following radiation. Curr. Neuropharmacol. 20 (5), 836–856. 10.2174/1570159x19666210809103346 34370636 PMC9881105

[B12] Finnerup N. B. Kuner R. Jensen T. S. (2021). Neuropathic pain: from mechanisms to treatment. Physiol. Rev. 101 (1), 259–301. 10.1152/physrev.00045.2019 32584191

[B13] Fonseca-Rodrigues D. Amorim D. Almeida A. Pinto-Ribeiro F. (2021). Emotional and cognitive impairments in the peripheral nerve chronic constriction injury model (CCI) of neuropathic pain: a systematic review. Behav. Brain Res. 399, 113008. 10.1016/j.bbr.2020.113008 33171146

[B14] Fu S. Rempson C. M. Puche V. Zhao B. Zhang F. (2022). Construction of disulfide containing redox-responsive polymeric nanomedicine. Methods 199, 67–79. 10.1016/j.ymeth.2021.12.011 34971759

[B15] García-Fernández A. Sancenón F. Martínez-Máñez R. (2021). Mesoporous silica nanoparticles for pulmonary drug delivery. Adv. Drug Deliv. Rev. 177, 113953. 10.1016/j.addr.2021.113953 34474094

[B16] Hassanzadeh K. Buccarello L. Dragotto J. Mohammadi A. Corbo M. Feligioni M. (2020). Obstacles against the marketing of curcumin as a drug. Int. J. Mol. Sci. 21 (18), 6619. 10.3390/ijms21186619 32927725 PMC7554750

[B17] Hegazy N. Rezq S. Fahmy A. (2020). Renin-angiotensin system blockade modulates both the peripheral and central components of neuropathic pain in rats: role of calcitonin gene-related peptide, substance P and nitric oxide. Basic Clin. Pharmacol. Toxicol. 127 (6), 451–460. 10.1111/bcpt.13453 32542932

[B18] Jang D. I. Lee A. H. Shin H. Y. Song H. R. Park J. H. Kang T. B. (2021). The role of tumor necrosis factor alpha (TNF-α) in autoimmune disease and current TNF-α inhibitors in therapeutics. Int. J. Mol. Sci. 22 (5), 2719. 10.3390/ijms22052719 33800290 PMC7962638

[B19] Jensen E. C. (2013). Quantitative analysis of histological staining and fluorescence using ImageJ. Anat. Rec. Hob. 296 (3), 378–381. 10.1002/ar.22641 23382140

[B20] Johanes A. Widayati R. Soedarsono N. Soegiharto B. M. (2020). Correlation between pain perception and CGRP expression during initial tooth alignment using either a self-ligating or a pre-adjusted bracket system. J. Contemp. Dent. Pract. 21 (12), 1312–1315. 10.5005/jp-journals-10024-2947 33893251

[B21] Kang S. A. Govindarajan R. (2021). Anti-calcitonin gene-related peptide monoclonal antibodies for neuropathic pain in patients with migraine headache. Muscle Nerve 63 (4), 563–567. 10.1002/mus.27153 33347632

[B22] Kong Z. L. Kuo H. P. Johnson A. Wu L. C. Chang K. L. B. (2019). Curcumin-loaded mesoporous silica nanoparticles markedly enhanced cytotoxicity in hepatocellular carcinoma cells. Int. J. Mol. Sci. 20 (12), 2918. 10.3390/ijms20122918 31207976 PMC6628080

[B23] Kotha R. R. Luthria D. L. (2019). Curcumin: biological, pharmaceutical, nutraceutical, and analytical aspects. Molecules 24 (16), 2930. 10.3390/molecules24162930 31412624 PMC6720683

[B24] Liang X. Wang Z. Wang S. Ruan F. Zhang Y. Shao D. (2025). Magnetic mesoporous silica nanoparticles loaded with peptides for the targeted repair of cavernous nerve injury underlying erectile dysfunction. Biomaterials 314, 122811. 10.1016/j.biomaterials.2024.122811 39265373

[B25] Liang Y. L. Belousoff M. J. Fletcher M. M. Zhang X. Khoshouei M. Deganutti G. (2020). Structure and dynamics of adrenomedullin receptors AM(1) and AM(2) reveal key mechanisms in the control of receptor phenotype by receptor activity-modifying proteins. ACS Pharmacol. Transl. Sci. 3 (2), 263–284. 10.1021/acsptsci.9b00080 32296767 PMC7155201

[B26] Lim E. Y. Lee C. Kim Y. T. (2022). The antinociceptive potential of camellia japonica leaf extract, (-)-Epicatechin, and rutin against chronic constriction injury-induced neuropathic pain in rats. Antioxidants (Basel) 11 (2), 410. 10.3390/antiox11020410 35204294 PMC8869459

[B27] Lin D. Xiao L. Qin W. Loy D. A. Wu Z. Chen H. (2022). Preparation, characterization and antioxidant properties of curcumin encapsulated chitosan/lignosulfonate micelles. Carbohydr. Polym. 281, 119080. 10.1016/j.carbpol.2021.119080 35074131

[B28] Liu C. Rokavec M. Huang Z. Hermeking H. (2023b). Curcumin activates a ROS/KEAP1/NRF2/miR-34a/b/c cascade to suppress colorectal cancer metastasis. Cell Death Differ. 30 (7), 1771–1785. 10.1038/s41418-023-01178-1 37210578 PMC10307888

[B29] Liu P. Zhang Y. Li X. Ma M. (2023a). DEAD-box helicase 54 regulates microglial inflammatory response in rats with chronic constriction injuries through NF-κB/NLRP3 signaling axis. J. Neurophysiol. 130 (2), 392–400. 10.1152/jn.00411.2022 37377223

[B30] Luo J. Chen H. Wang G. Lyu J. Liu Y. Lin S. (2023). CGRP-loaded porous microspheres protect BMSCs for alveolar bone regeneration in the periodontitis microenvironment. Adv. Healthc. Mater 12 (28), e2301366. 10.1002/adhm.202301366 37515813

[B31] Macone A. Otis J. a. D. (2018). Neuropathic pain. Semin. Neurol. 38 (6), 644–653. 10.1055/s-0038-1673679 30522140

[B32] Martin S. L. Reid A. J. Verkhratsky A. Magnaghi V. Faroni A. (2019). Gene expression changes in dorsal root ganglia following peripheral nerve injury: roles in inflammation, cell death and nociception. Neural Regen. Res. 14 (6), 939–947. 10.4103/1673-5374.250566 30761997 PMC6404509

[B33] Paige C. Plasencia-Fernandez I. Kume M. Papalampropoulou-Tsiridou M. Lorenzo L. E. David E. T. (2022). A female-specific role for calcitonin gene-related peptide (CGRP) in rodent pain models. J. Neurosci. 42 (10), 1930–1944. 10.1523/jneurosci.1137-21.2022 35058371 PMC8916765

[B34] Park J. Fong P. M. Lu J. Russell K. S. Booth C. J. Saltzman W. M. (2009). PEGylated PLGA nanoparticles for the improved delivery of doxorubicin. Nanomedicine. 5 (4), 410–418. 10.1016/j.nano.2009.02.002 19341815 PMC2789916

[B35] Park S. Cho B. B. Anusha J. R. Jung S. Justin Raj C. Kim B. C. (2020). Synthesis of (64)Cu-radiolabeled folate-conjugated iron oxide nanoparticles for cancer diagnosis. J. Nanosci. Nanotechnol. 20 (4), 2040–2044. 10.1166/jnn.2020.17205 31492210

[B36] Polyak B. Friedman G. (2009). Magnetic targeting for site-specific drug delivery: applications and clinical potential. Expert Opin. Drug Deliv. 6 (1), 53–70. 10.1517/17425240802662795 19236208

[B37] Rosenberger D. C. Blechschmidt V. Timmerman H. Wolff A. Treede R. D. (2020). Challenges of neuropathic pain: focus on diabetic neuropathy. J. Neural Transm. (Vienna) 127 (4), 589–624. 10.1007/s00702-020-02145-7 32036431 PMC7148276

[B39] Sun Y. Wang Z. Zhang P. Wang J. Chen Y. Yin C. (2020). Mesoporous silica integrated with Fe(3)O(4) and palmitoyl ascorbate as a new nano-Fenton reactor for amplified tumor oxidation therapy. Biomater. Sci. 8 (24), 7154–7165. 10.1039/d0bm01738h 33155581

[B40] Uddin S. J. Hasan M. F. Afroz M. Sarker D. K. Rouf R. Islam M. T. (2021). Curcumin and its multi-target function against pain and inflammation: an update of pre-clinical data. Curr. Drug Targets 22 (6), 656–671. 10.2174/1389450121666200925150022 32981501

[B41] Uribe Madrid S. I. Pal U. Kang Y. S. Kim J. Kwon H. Kim J. (2015). Fabrication of Fe3O4@mSiO2 core-shell composite nanoparticles for drug delivery applications. Nanoscale Res. Lett. 10, 217. 10.1186/s11671-015-0920-5 26034415 PMC4444644

[B42] Wang Y. Cheng Q. Liu J. Tariq Z. Zheng Z. Li G. (2020). Tuning microcapsule shell thickness and structure with silk fibroin and nanoparticles for sustained release. ACS Biomater. Sci. Eng. 6 (8), 4583–4594. 10.1021/acsbiomaterials.0c00835 33455196

[B38] Xiao L. Ding M. Fernandez A. Zhao P. Jin L. Li X.(2017). Curcumin alleviates lumbar radiculopathy by reducing neuroinflammation, oxidative stress and nociceptive factors. Eur. Cell Mater. 33: 279–293. 10.22203/eCM.v033a21 28485773 PMC5521990

[B43] Xu Q. Niu C. Li J. Hu C. He M. Qiu X. (2022). Electroacupuncture alleviates neuropathic pain caused by spared nerve injury by promoting AMPK/mTOR-mediated autophagy in dorsal root ganglion macrophage. Ann. Transl. Med. 10 (24), 1341. 10.21037/atm-22-5920 36660615 PMC9843338

[B44] Yadav Y. C. Pattnaik S. Swain K. (2019). Curcumin loaded mesoporous silica nanoparticles: assessment of bioavailability and cardioprotective effect. Drug Dev. Ind. Pharm. 45 (12), 1889–1895. 10.1080/03639045.2019.1672717 31549866

[B45] Zhang G. Liu N. Zhu C. Ma L. Yang J. Du J. (2019). Antinociceptive effect of isoorientin against neuropathic pain induced by the chronic constriction injury of the sciatic nerve in mice. Int. Immunopharmacol. 75, 105753. 10.1016/j.intimp.2019.105753 31336334

[B46] Zhang N. Jia C. Ma X. Li J. Wang S. Yue B. (2021). Hierarchical core-shell Fe_3_O_4_@mSiO_2_@Chitosan nanoparticles for pH-responsive drug delivery. J. Nanosci. Nanotechnol. 21 (5), 3020–3027. 10.1166/jnn.2021.19154 33653475

[B47] Zhao S. Yu X. Qian Y. Chen W. Shen J. (2020). Multifunctional magnetic iron oxide nanoparticles: an advanced platform for cancer theranostics. Theranostics 10 (14), 6278–6309. 10.7150/thno.42564 32483453 PMC7255022

[B48] Zhou C. Shi X. Huang H. Zhu Y. Wu Y. (2014). Montelukast attenuates neuropathic pain through inhibiting p38 mitogen-activated protein kinase and nuclear factor-kappa B in a rat model of chronic constriction injury. Anesth. Analg. 118 (5), 1090–1096. 10.1213/ane.0000000000000174 24686047

