# Supplementary material for: Mesoporous silica loaded with calcitonin gene-related peptide antagonist and curcumin alleviate oxidative stress and inflammation in the sciatic nerve
Source: Front Mol Biosci. 2025 Mar 24;12:1510141. doi: 10.3389/fmolb.2025.1510141 (PMC11973289; doi:10.3389/fmolb.2025.1510141)
Supplement: Supplementary file 1 [file Supplementaryfile1.docx]

Supplementary Material

# Supplementary Figures


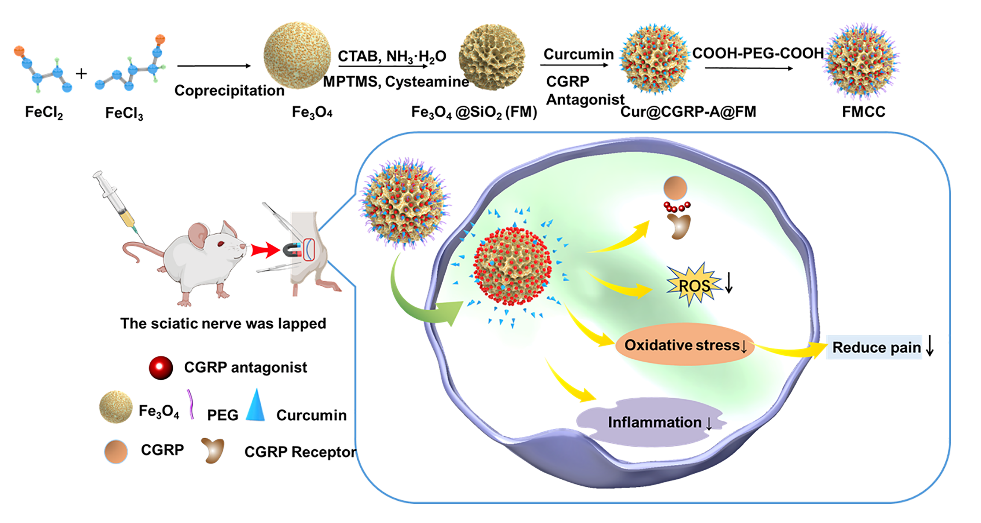


**Supplementary Figure 1**. Schematic diagram of preparation of FMCC and of in vitro and in vivo experiments.


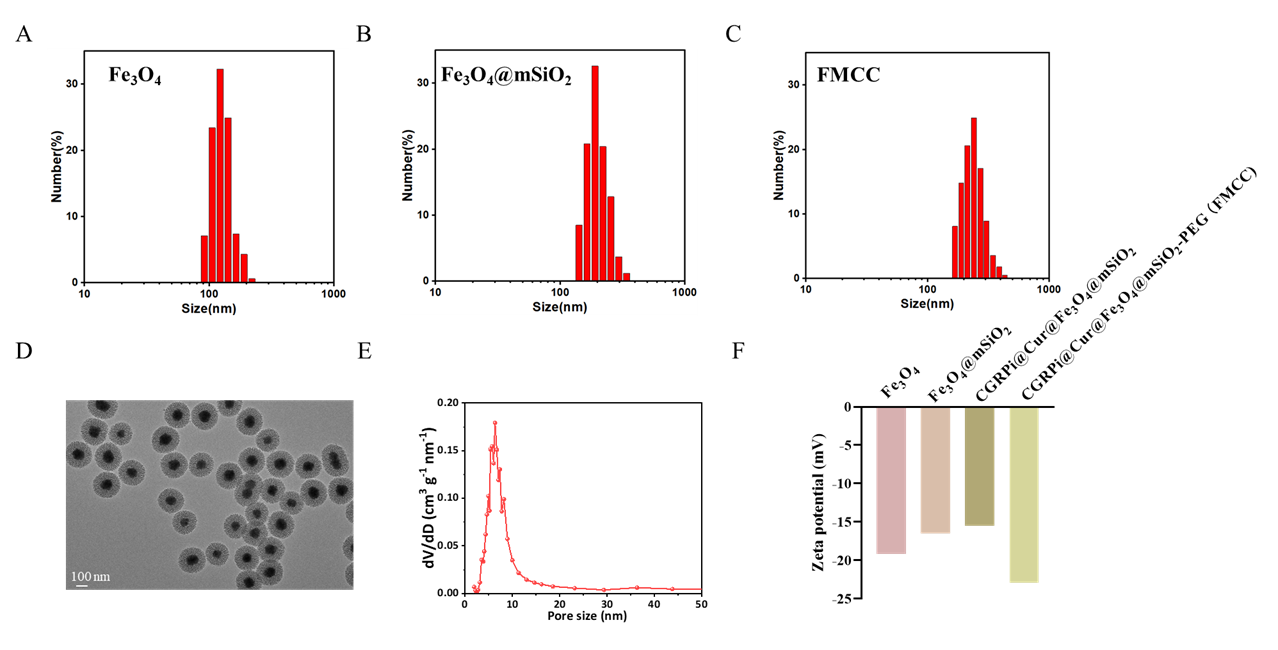


**Supplementary Figure 2.** (A-C) Fe_3_O_4_ (A), Fe_3_O_4_@mSiO_2_ (B), and FMCC (C) particle size distribution of DLS detection; (D) TEM images of FMCC nanoparticles; (E) Pore size of FMCC nanoparticles; (F) Zeta potential data of Fe_3_O_4_, Fe_3_O_4_@mSiO_2_, CGRP-A@*Cur*@Fe_3_O_4_@mSiO_2_ and CGRP-A@*Cur*@Fe_3_O_4_@mSiO_2_-PEG (FMCC).

**Supplementary Figure 3.** Quantitative analysis of corresponding mean fluorescence intensity in Figure 2E.

**Supplementary Figure 4.** (A) RT-PCR detection of CGRP mRNA expression levels in BV2 cells after LPS, CGRP-A@FM, *Cur*@FM, and FMCC treatment.


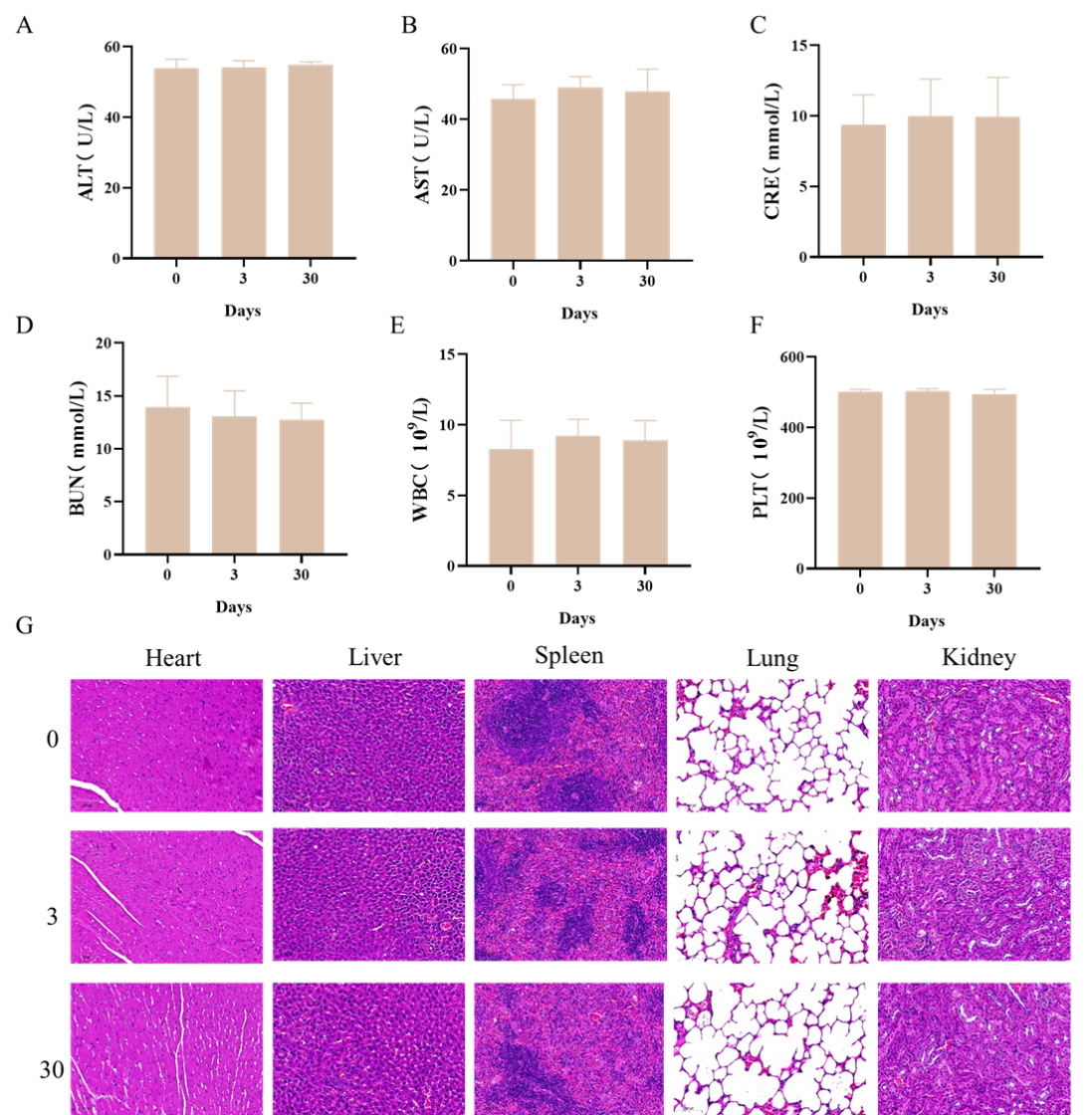


**Supplementary Figure 5**. (A-F) ALT, AST, CRE, BUN, WBC, and PLT detection; (G) H&E staining of important organs and tissues.
